# A Mouse Model of Dilated Cardiomyopathy Produced by Isoproterenol Acute Exposure Followed by 5-Fluorouracil Administration

**DOI:** 10.3390/jcdd10060225

**Published:** 2023-05-23

**Authors:** Nadia Salerno, Mariangela Scalise, Fabiola Marino, Andrea Filardo, Antonio Chiefalo, Giuseppe Panuccio, Michele Torella, Antonella De Angelis, Salvatore De Rosa, Georgina M. Ellison-Hughes, Konrad Urbanek, Giuseppe Viglietto, Daniele Torella, Eleonora Cianflone

**Affiliations:** 1 Department of Experimental and Clinical Medicine, Magna Graecia University, 88100 Catanzaro, Italy; nadia.salerno@unicz.it (N.S.); m.scalise@unicz.it (M.S.); marino@unicz.it (F.M.); antonio.chiefalo@unicz.it (A.C.); viglietto@unicz.it (G.V.); 2 Department of Medical and Surgical Sciences, Magna Graecia University, 88100 Catanzaro, Italy; filardo@unicz.it (A.F.); panuccio@unicz.it (G.P.); saderosa@unicz.it (S.D.R.); 3 Department of Experimental Medicine, University of Campania “L. Vanvitelli”, 80138 Naples, Italy; michele.torella@unicampania.it (M.T.); antonella.deangelis@unicampania.it (A.D.A.); 4 Centre for Human and Applied Physiological Sciences, School of Basic and Medical Biosciences, Faculty of Life Sciences & Medicine, King’s College London, Guy’s Campus, London SE1 1UL, UK; georgina.ellison@kcl.ac.uk; 5 Department of Molecular Medicine and Medical Biotechnology, Federico II University, 88121 Naples, Italy; konradarkadiusz.urbanek@unina.it

**Keywords:** dilated cardiomyopathy, cardiac fibrosis, cardiac inflammation, cardiac senescence

## Abstract

Appropriate dilated cardiomyopathy (DCM) animal models are highly desirable considering the pathophysiological and clinical heterogeneity of DCM. Genetically modified mice are the most widely and intensively utilized research animals for DCM. However, to translate discoveries from basic science into new and personalized medical applications, research in non-genetically based DCM models remains a key issue. Here, we characterized a mouse model of non-ischemic DCM induced by a stepwise pharmacologic regime of Isoproterenol (ISO) high dose bolus followed by a low dose systemic injection of the chemotherapy agent, 5-Fluorouracil (5-FU). C57BL/6J mice were injected with ISO and, 3 days after, were randomly assigned to saline or 5-FU. Echocardiography and a strain analysis show that ISO + 5FU in mice induces progressive left ventricular (LV) dilation and reduced systolic function, along with diastolic dysfunction and a persistent global cardiac contractility depression through 56 days. While mice treated with ISO alone recover anatomically and functionally, ISO + 5-FU causes persistent cardiomyocyte death, ensuing in cardiomyocyte hypertrophy through 56 days. ISO + 5-FU-dependent damage was accompanied by significant myocardial disarray and fibrosis along with exaggerated oxidative stress, tissue inflammation and premature cell senescence accumulation. In conclusions, a combination of ISO + 5FU produces anatomical, histological and functional cardiac alterations typical of DCM, representing a widely available, affordable, and reproducible mouse model of this cardiomyopathy.

## 1. Introduction

Heart failure (HF) is a leading cause of morbidity and mortality in developed countries. Despite several important therapeutic advances for the treatment of symptomatic HF, the prevalence, mortality, and cost associated with HF continue to grow [1,2,3]. A left ventricular (LV) ejection fraction (EF) remains the major parameter for diagnosis, phenotyping, prognosis, and treatment decisions in HF. On that basis, HF has been categorized in HF with preserved, mildly-reduced, and reduced EF [4]. The treatment of patients with reduced EF depends on, at least in part, the identification of the underlying disease process. One of the primary diagnostic issues is on differentiating an underlying cause for the LV dysfunction that is related to dilated cardiomyopathy (DCM) or coronary artery disease (CAD). While for CAD a significant number of therapeutic approaches exist, for DCM, there is a need to develop novel preventative and reparative therapies [5]. The development of these novel therapies requires testing the putative therapeutic strategies in appropriate DCM animal models [6].

The ideal animal model should be able to reproduce the typical anatomical, functional, and histological features of the desired type of cardiomyopathy. Reflecting the higher prevalence of left-sided heart failure in the general population, LV dysfunction models of DCM are more widely employed. Human DCM is defined as a spectrum of myocardial diseases that share ventricular dilatation and depressed contractility [5]. Ischemic cardiomyopathy is the most common type of DCM. On the other hand, primary DCM is a non-ischemic heart muscle disease with diverse etiologies, including genetic mutations, infections, inflammation, autoimmune diseases, exposure to toxins and endocrine or neuromuscular causes [5,6]. Idiopathic and familial disease are the most reported causes of primary DCM [5,6]. Mutations in several genes cause DCM, including genes encoding structural components of the sarcomere and desmosome [5]. Nongenetic forms of DCM can result from different etiologies, including the inflammation of the myocardium due to a (mostly viral) infection; exposure to drugs, toxins, or allergens; and systemic endocrine or autoimmune diseases [5]. The DCM anatomic and functional phenotype is mainly characterized by a progressive LV dilatation, together with a ventricular shape transition from its original ellipsoid shape to a more spherical one, wall thinning, and a global reduction in contractility, which entails a decrease in stroke volume and cardiac index and increased strain parameters [5,6,7]. From a histology perspective, DCM is mainly characterized by myocardial disarray, fibrosis, cell death, cardiomyocyte hypertrophy, scar formation, and inflammatory infiltration [5,6,7]. Because of the slow progression of the disease, DCM is often detected only at an end stage when the scar tissue is well established and is refractory to medication to reverse the process. Therefore, new therapeutic opportunities are highly searched for.

Genetically modified mice are the most widely and intensively utilized research animals and allowed high throughput studies on non-ischemic DCM [3]. However, to translate discoveries from basic science into medical applications, research in non-genetically based DCM models remains a key issue. On this premise, here, we describe a mouse model of non-ischemic DCM induced by a stepwise pharmacologic regime of Isoproterenol high dose bolus followed by a low dose systemic injection of the chemotherapy agent, 5-FU. This combination produced anatomical, histological, and functional cardiac alterations typical of DCM, representing a widely available, affordable, and reproducible animal model of this cardiomyopathy.

## 2. Materials and Methods

### 2.1. Animals

#### 2.1.1. Origin of the Animals

Male C57BL/6J mice that were 12/14-weeks-old were used in the study (stock number 000664, Jackson Labs, Bar Harbor, ME, USA).

#### 2.1.2. Approval of the Ethics Committees

All animal experimental procedures were approved by Magna Graecia Institutional Review Boards on Animal Use and Welfare and performed according to the Guide for the Care and Use of Laboratory Animals from directive 2010/63/EU of the European Parliament.

#### 2.1.3. Conditions of Maintenance

All animals received human care, and all efforts were made to minimize animal suffering. Animals were euthanized by pentobarbital overdosage. Mice were housed under controlled conditions of 25 °C, 50% relative humidity, and a 12-h light (6:00–18:00) and 12-h dark cycle with water and food (containing 18.5% protein) available ad libitum.

#### 2.1.4. Formation of Study Groups

Before each invasive procedure, mice were anesthetized by intraperitoneal injection (i.p) of ketamine (100 mg/kg) and xylazine (5 mg/kg) or inhaled isoflurane (isoflurane 1.5% oxygen 98.5%, Iso-Vet, Healthcare). The 12/14-week-old C57BL/6J male mice were randomly assigned to receive ISO at 200 mg/Kg (n = 18) injected subcutaneously under the inter-scapular skin. Two mice acutely died within 2 days after ISO. Additional sex and age-matched mice (n = 8) were injected with an equal volume of saline representing controls (CTRL). Three days after ISO, the surviving mice (n = 16) were randomly assigned to saline (n = 8) or 5-FU administration (15 mg/kg/day, n = 8) through mini-osmotic pumps (Alzet, model 2004, DURECT Corporation, Cupertino, CA 95014, USA) inserted under the infra-scapular skin for 28 days.

### 2.2. Drug Administration

Isoproterenol (ISO, Sigma-Aldrich #I6504; St. Louis, MO, USA) was prepared by dissolving a desired amount of powder in NaCl 0.9% [8,9,10]. The solution obtained was protected from the light and kept on ice until the injections. Before any ISO/Saline injection, the body weight of animals was determined, mice were anesthetized using isoflurane, and baseline echocardiography was obtained. Then, mice were randomly divided in the different groups and, on awakening, prepared to receive saline or ISO at dose of 200 mg/Kg [9,10]. The solutions were injected subcutaneously under the inter-scapular skin. All ISO injections were administered to male 12/14-weeks-old mice. After 72 h of receiving ISO at dose of 200 mg/Kg, mice received systemic administration of 5-FU (15 mg/Kg/day) for 25 days through subcutaneously implantation of mini-osmotic pumps [9,10]. Before pumps implantation, the mice were anesthetized using isoflurane. Then, 28 days after ISO injection, 5-FU-realising mini-pumps were removed. The animals were sacrificed after additional 28 days, and the hearts were fixed in 10% buffered formalin or in 4% paraformaldehyde (PFA) for immunohistochemistry analysis or dissociated to obtain a cardiac cell preparation.

### 2.3. Echocardiography

Echocardiography was performed as previously reported and validated in our lab [8,9,10]. Prior to echocardiography, mice were anesthetized with isoflurane. Unconscious mice were weighed and secured in a supine position on a temperature-controlled restraining board. Anesthesia was maintained with 1–2% isoflurane in oxygen delivered through a nose cone. Four-limb lead electrocardiograms (Vevo3100 and MP150, Biopac, Goleta, CA, USA) were simultaneously recorded. All hair in the thoracic region was removed using a depilatory agent, and the area was cleaned with water. Ultrasound gel was applied to the thoracic region to improve sound wave transmission. All mice were maintained at heart rates > 400 bpm while images were recorded. Echocardiographic images were obtained with a Vevo 3100 system (Visualsonics, Inc., Toronto, ON, Canada) equipped with a MX550D ultra-high frequency linear array transducer (22–55 MHz). The transducer was positioned in a stationary stand perpendicular to the mouse (in some cases, manual adaptations were needed for optimal imaging). In brief, a frame rate of >200 frames per minute was maintained for all B-mode and M-mode images. B-mode long-axis parasternal images were recorded when optimal views of the aorta, papillary muscle, and endocardium were visible. M-mode short-axis images were recorded at the level of the papillary muscles and the LV was bisected to obtain the optimal M-mode selection. Conventional echocardiographic measurements of the LV included ejection fraction (EF), fractional shortening (FS), end-diastolic dimension (EDD), end-systolic dimension (ESD), anterior and posterior wall thickness, and mass were obtained. For long-axis B-mode measurements, the endocardium was traced semi automatically beginning from the mitral valve and excluding the papillary muscle. EF and FS were calculated by software using standard computational methods. The apical 4-chamber view was acquired to assess mitral and tricuspid regurgitation. Advanced cardiac analysis (regional and global cardiac measurements) were assessed by speckle-tracking echocardiography (Vevo LAB analysis software; VisualSonics, Toronto, ON, Canada). Cardiac cycles were acquired digitally from the parasternal long-axis and mid-ventricular short-axis views for the assessment of radial, circumferential, and longitudinal systolic strain/velocity (in accordance with myocardial fiber orientation at varying levels of the LV wall) and time-to-peak systolic strain/velocity. Images selected for strain analysis had well-defined endocardium and epicardium borders and no substantial image artefacts. Image analysis was performed according to manufacturer’s instructions. The endocardium and epicardium were traced semi-automatically using VevoStrain software. The traces were manually adjusted to ensure adequate tracking of endocardium and epicardium borders. Velocity, displacement, strain, and strain rate were calculated for radial and longitudinal planes. In long-axis, the basal anterior-septum, mid-anterior-septum, apical anterior-septum, basal posterior wall, mid-posterior wall, and apical posterior segments were defined. In mid-ventricular short-axis, the anterior, anterior-septum, inferior-septum, inferior, posterior, and anterior-lateral segments were further delineated. Tissue contraction patterns were expressed as negative strain values for longitudinal and circumferential motion and positive values for radial strain. In each segment, peak systolic strain (%) and time-to-peak systolic strain (ms) were analysed. Global average peak values for circumferential and longitudinal strain are reported.

All measurements were performed offline using the VevoLab Analysis Software (FUJIFILM VisualSonics Inc., Toronto, ON, Canada).

### 2.4. Myocyte Necrosis Analysis

To assess cardiomyocyte necrosis, 12/14-weeks-old mice received intraperitoneal injection of 100 µg/100 µL of a monoclonal antibody against cardiac myosin (MF-20, ID: AB_2147781, DSHB, Iowa City, IA, USA) two hours before sacrifice [10], and the hearts were fixed in 4% PFA for immunohistochemistry analysis. The primary antibody was revealed by respective anti-mouse IgG secondary antibody (1:100 dilution; Jackson Immunoresearch, Ely, Cambridgeshire, UK).

### 2.5. Mouse Cardiomyocytes Isolation

A calcium-free solution was used to digest the hearts and prepared as follows: sodium chloride at a final concentration of 126 mM; glucose at a final concentration of 22 mM; HEPES at a final concentration of 24 mM; potassium chloride at a final concentration of 4.4 mM; magnesium dichloride at a final concentration of 5 mM; creatine at a final concentration of 5 mM; taurine at a final concentration of 20 mM; sodium pyruvate at a final concentration of 5 mM; and sodium dihydrogen phosphate at a final concentration of 1 mM. To increase cardiomyocytes yield, 2,3-butanedione monoxime 10 mM was added to the solution. The solution was filtered through a 0.22 µm-pore filter into a sterile container and stored at 4 °C for up to 1 week. For each heart isolation, a total amount of 50 mL of buffer was used. The chest of anesthetized mice was excised, the aorta cannulated and hung on a retrograde perfusion system (Langendorff method), then perfused with enzyme-containing solutions as previously described [9,11]. Briefly, the cannulated hearts were first perfused with the calcium-free solution, followed by type II collagenase digestion in presence of Ca^2+^ 0.1 mM and then washed with a Ca^2+^ 0.1 mM solution. When perfusion was completed, the hearts were taken down from the cannula, cut into small pieces, and gently triturated with a pipette. To enrich the cell suspension with viable cardiac myocytes and to remove large or undigested tissue, preparations were filtered through a 100 µm cell strainer and then centrifuged to separate cardiomyocytes from other cardiac cell types. Viable cardiomyocytes were then allowed to sediment with gravity. The sedimentation procedure was repeated several times [11,12].

### 2.6. Tissue Harvesting, Histology, and Immunohistochemistry

For immunohistochemistry analysis, the abdominal aorta was cannulated and the heart arrested in diastole using cadmium chloride/potassium solution. The animals were perfused through the cannulated aorta and fixed with 10% buffered formalin or with 4% PFA. The hearts were cut into apical, mid, and basal regions and right and left atria. After being weighed, the LV was sectioned and embedded in paraffin or in Optimal Cutting Temperature Compound (O.C.T.). Tissues were cut in 5 µm or 10 µm cross-sections, respectively. Sections were stained with Hematoxylin and Eosin (H&E, BioOptica, Milan, Italy) following standard procedures [13]. Formalin-fixed sections were stained with Picrosirius Red Staining (Bio Optica, Milan, Italy) for bioquantification of fibrosis [14]. Senescent cardiac cells and cardiomyocytes were detected using the primary antibody anti-p16 (1:100 dilution; Rockland, Philadelphia, PA, USA). The positive reactions were visualized using a Labelled Polymer-HRP complex and 3,3′-diaminobenzidine tetrahydrochloride (DAB) chromogen (EnVision+ Dual Link System-HRP, DAKO, Santa Clara, CA, USA). Sections were then counterstained with Hematoxylin and examined with light microscopy (DMI3000B, Leica Microsystems, Wetzlar, Germany). Cardiomyocyte cross-sectional area was measured through immunostaining with Wheat Germ Agglutinin Alexa Fluor 594-conjugate, WGA (1:200 dilution; ThermoFisher Scientific, Waltham, MA, USA) and digital analysis of acquired cardiac cross-section images (1128 LAS AF Software, Leica Microsystems, Wetzlar, Germany). Cardiomyocyte diameter was measured across the nucleus on 3 transverse sections (~500 myocytes/animal were sampled). For Reactive Oxygen Species (ROS) detection, the Dihydroethidium (DHE) Assay Kit (Abcam, Cambridge, UK) and a FITC-conjugated Anti-Nitrotyrosine (3-NT) antibody (1:100, Abcam, Cambridge, UK) were used according to slight modifications of manufacturer’s instructions [11,14]. Apoptotic cells were detected using a FITC-conjugated Anti-Terminal deoxynucleotidyl transferase kit (In Situ Cell Death Detection Kit, Fluorescein, Sigma-Aldrich, St. Louis, MO, USA). For immunofluorescent staining, the following primary antibodies were used: anti-Cardiac Troponin I (1:100 dilution; Abcam, Cambridge, UK) and anti-CD45 (1:50 dilution; Rockland, Philadelphia, PA, USA). The specific primary antibodies were revealed by respective anti-rabbit IgG or anti-goat IgG secondary antibody (1:100 dilution; Jackson Immunoresearch, Ely, Cambridgeshire, UK). The nuclei were counterstained with the DNA binding dye, 4, 6-diamidino-2-phenylindole (DAPI, Sigma-Aldrich, St. Louis, MO, USA) at 1 µg/mL. The number of inflammatory cells was expressed per mm^2^. The number of p16 positive cardiac cells and cardiomyocytes was expressed as a percent fraction of the total cardiomyocyte number per mm^2^. The number of necrotic/dead MF-20^pos^ cardiomyocytes was manually counted in cardiac cross sections for each power field using a 63× objective for a total of 20 fields [10], and the number of MF-20^pos^ cardiomyocytes was expressed as a percent fraction of the total cardiomyocyte number per mm^2^ [10]. Unless otherwise specified, all stainings were acquired and analysed using confocal microscopy (LEICA TCS SP5 and SP8, Leica Microsystems, Wetzlar, Germany).

### 2.7. Quantitative RT-PCR (qPCR)

RNA was extracted from CMs using the TRIzol Reagent (Ambion, Waltham, Ma, USA) and quantified using a Nanodrop 2000 Spectrophotometer (Thermo Fisher Scientific, Waltham, MA, USA). Reverse transcription was performed with 0.5–1 µg of RNA, using the HighCapacity cDNA Kit (Applied Biosystems, Waltham, MA, USA). Quantitative qPCR was performed using the following TaqMan Primers (Applied Biosystems, Ebersberg, Germany) [9,10,11,12]: Gapdh ID: Mm99999915_g1; Myh7 ID: Mm00600555_m1; Nppa ID: Mm01255747_g1; Bnp ID: Mm01255770_g1, using the StepOne Plus Real-Time PCR System (Applied Biosystems, Waltham, MA, USA). All reactions were carried out in triplicate.

### 2.8. Statistical Analysis

Statistical analysis was performed with GraphPad Prism version 9.00 for Macintosh (GraphPad Software). For comparison between multiple groups, ANOVA was used. A *p* value < 0.05 was considered significant. Tukey’s post hoc method was used to locate the differences. In these cases, the Type 1 error (α = 0.05) was corrected by the number of statistical comparisons performed.

## 3. Results

### 3.1. Echocardiography Analysis Reveals Typical Features of Dilated Cardiomyopathy Resulting from Acute Isoproterenol Exposure Followed by 5-Fluorouracil Administration

**Figure 1:**
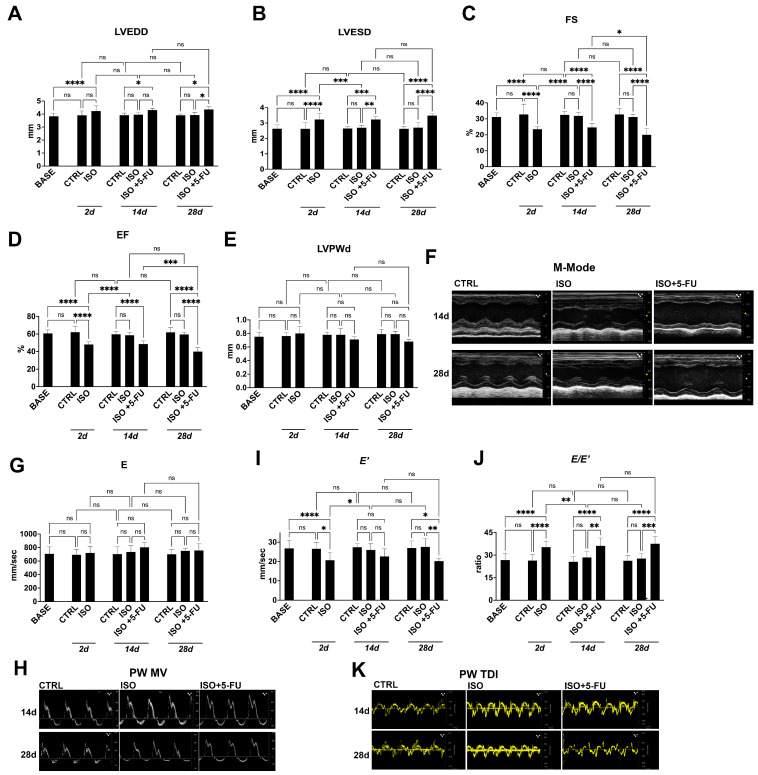
*Echocardiography analysis reveals typical features of DCM resulting from acute Isoproterenol exposure followed by 5–Fluorouracil administration.* (**A**–**F**) Cumulative and representative data of cardiac dimensions and function in the different groups and time points of the study. (**A**), LVEDD = left ventricular end-diastolic diameter; (**B**) LVESD = left ventricular end-systolic diameter; (**C**) EF = ejection fraction; (**D**), FS = fractional shortening; (**E**), LVPWd = left ventricular posterior wall thickness at end diastole. (**F**) representative M-mode tracing of long-axis left ventricle. * *p* < 0.001; ** *p* < 0.0001. (**G**,**H**) Cumulative data (**G**) and representative pulsed-wave (PW) Doppler mitral velocity (MV) tracing (**H**) assessing diastolic function. * *p* < 0.05; ** *p* < 0.01; *** *p* < 0.001; **** *p* < 0.0001. (**I**–**K**) Cumulative data of diastolic function (E′ and E/E′ ratio, respectively I and J) and relative representative PW tissue Doppler imaging (TDI) velocity tracing (**K**). * *p* < 0.05; ** *p* < 0.01; *** *p* < 0.001; **** *p* < 0.0001. BASE = baseline (n = 26); CTRL: saline treated control animals (n = 8); ISO: isoproterenol treated mice (n = 18 at 2 days; n = 8 at 14 and 28 days); ISO + 5-FU: isoproterenol plus 5-Fluorouracil treated mice (n = 8). Data are mean ± SD. ns, no significance.

Here, to assess that the ISO + 5-FU-induced myocardial damage and dysfunction [8,9,10,15] represented in a reliable model of DCM, 12/14-week-old C57BL/6J male mice were randomly assigned to receive ISO at 200 mg/Kg (n = 18) injected subcutaneously under the inter-scapular skin. Additional sex and age-matched mice (n = 8) were injected with an equal volume of saline representing controls (CTRL). Two mice acutely died within 2 days after ISO. Two days after ISO, echocardiography show an increased LV end systolic diameter (LVESD) as compared to baseline, owing to EF and FS depression (~20% and ~25% respectively) (Figure 1A–F, Table 1). Diastolic dysfunction was also evident 2 days after ISO when compared to baseline, as shown by a significant decrease in the E′ value (indicating a reduction of LV longitudinal myocardial relaxation) (Figure 1G–K, Table 1) and a significant increase in the E/E′ (the ratio of trans-mitral Doppler early filling velocity to tissue Doppler early diastolic mitral annular velocity, an index of LV end-diastolic filling pressure) (Figure 1G–K, Table 1).

Three days after ISO, the surviving mice (n = 16) were randomly assigned to saline (n = 8) or 5-FU (15 mg/kg/day, n = 8) through mini-osmotic pumps inserted under the infra-scapular skin. Mice treated with ISO and saline (ISO) at 14 days through 28 days had returned their systolic and diastolic function to baseline, indistinguishable from saline-treated CTRL mice (Figure 1A–K, Table 1). On the other hand, mice receiving 5-FU administration already at 14 days during treatment show an increased LV end-systolic diameter (LVESD) and LV end-diastolic diameters (LVEDD) (~22% and ~9% respectively) as compared to CTRL and ISO, owing to LV EF and FS depression (~19% and ~24% respectively, Figure 1A–F, Table 1). Diastolic dysfunction was also evident in ISO + 5-FU mice when compared to CTRL and ISO + saline, as shown by a significant increase in the E/E′ (~41%, Figure 1J, Table 1). All these systolic and diastolic parameters were persistently altered at 28 days in ISO + 5-FU mice, showing evident sisto-diastolic dysfunction as compared to CTRL and ISO mice (Figure 1A–K, Table 1).

These results demonstrate that ISO + 5FU in mice induces progressive ventricular dilation and reduced systolic function, along with diastolic dysfunction, reminiscent of a typical dilated cardiomyopathy.

### 3.2. Strain Analysis Reveals Typical Features of Dilated Cardiomyopathy Resulting from Isoproterenol + 5-Fluorouracil Administration

**Figure 2:**
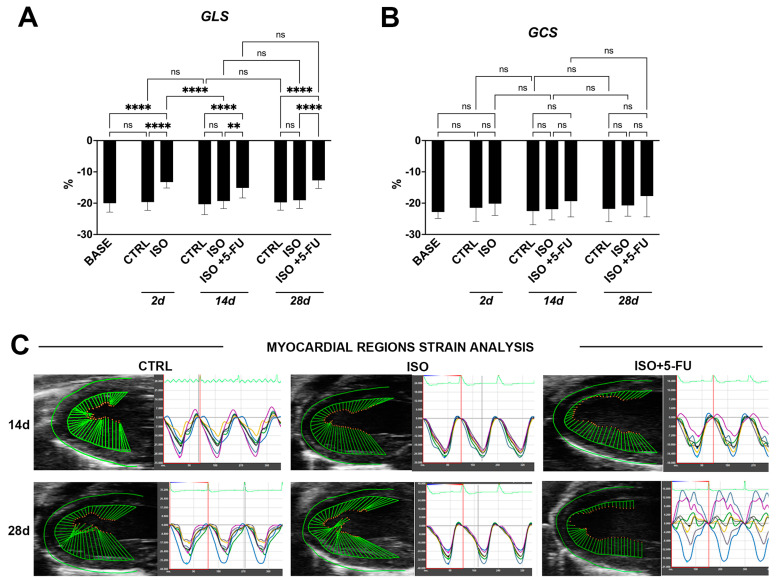
*Strain Analysis Reveals Typical Features of DCM resulting from Isoproterenol + 5-Fluorouracil administration.* (**A**–**C**) Cumulative and representative data of strain analysis in the different groups and time points of the study. (**A**) GLS = global longitudinal strain; (**B**) GCS = global circumferential strain; (**C**) Representative strain tracing of long-axis left ventricle. ** *p* < 0.01. **** *p* < 0.0001. BASE = baseline (n = 26); CTRL: saline treated control animals (n = 8); ISO: isoproterenol treated mice (n = 18 at 2 days; n = 8 at 14 and 28 days); ISO + 5FU: isoproterenol plus 5FU treated mice (n = 8). Data are mean ± SD. ns, no significance.

To directly measure myocardial contractility and the effects of ISO + 5FU on global and regional myocardial function, a speckle-tracking based strain analysis on the long axis and short axis B-mode was performed [10,11]. ISO significantly decreased the value of the global longitudinal strain (GLS), ~32%, at 2 days compared to the CTRL (Figure 2A, Table 1). At same time point, the global circumferential strain (GCS) did not change significantly after ISO compared to the CTRL (Figure 2B, Table 1). Global and regional strain analysis values returned to normal at 14 days through 28 days (Figure 2A,B). On the other hand, GLS but not GCS worsened through 28 days in ISO + 5-FU mice, and GLS was significantly altered when compared to the CTRL and ISO (~33% reduction, Figure 2A–C, Table 1).

Furthermore, the regional speckle-tracking strain analysis shows an increase in the time to peak value in all six segments together with wall motion abnormalities and wall synchronicity alterations through 28 days (Figure 2C, Table 1)

### 3.3. Dilated Cardiomyopathy Following ISO+ 5-FU Is Persistent at Long Follow-Up

We then investigated whether the DCM functional phenotype in ISO + 5-FU mice persisted over a longer time point and at 56 days after primary damage by ISO.

**Figure 3:**
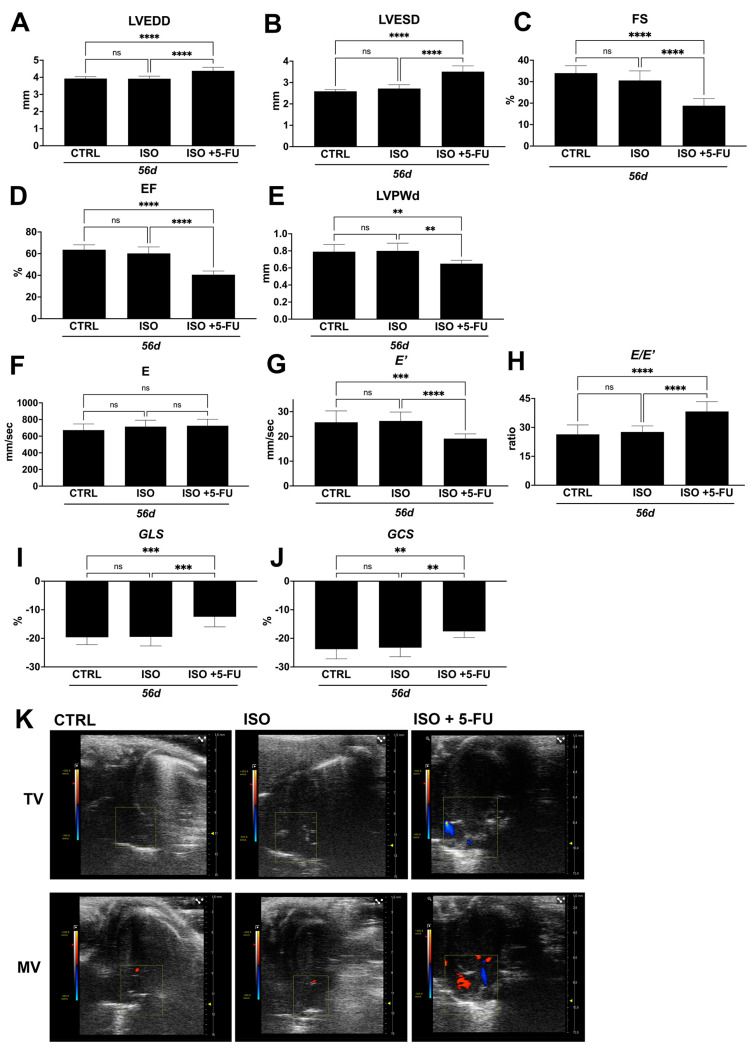
*Dilated Cardiomyopathy following ISO+ 5–FU is persistent at long follow-up.* (**A**–**E**) Cumulative and representative data of cardiac dimensions and function in the different groups at 56 days. (**A**) LVEDD = left ventricular end-diastolic diameter; (**B**) LVESD = left ventricular end-systolic diameter; (**C**) FS = fractional shortening; (**D**) EF = ejection fraction; (**E**) LVPWd = left ventricular posterior wall thickness at end diastole. ** *p* < 0.01; **** *p* < 0.0001. (**F**–**H**) Cumulative diastolic data at 56 days. *** *p* < 0.001; **** *p* < 0.0001. (**I**,**J**) Cumulative strain analysis data at 56 days. *** *p* < 0.001; ** *p* < 0.01. (**K**) Representative mitral and tricuspid valve color Doppler analysis. CTRL: saline treated control animals (n = 8); ISO: isoproterenol treated mice (n = 8); ISO + 5-FU: isoproterenol plus 5-FU treated mice (n = 8). Data are mean ± SD. ns, no significance.

LVEDD, LVESD, EF, and FS were consistently altered from 28 through 56 days in ISO + 5-FU mice (Figure 3A–D, Table 2). These standard echocardiographic parameters were all significantly affected in ISO + 5-FU mice when compared to ISO and CTRL animals at this longer time point (Figure 3A–D, Table 2). Moreover, an M-mode analysis showed a significant reduction in left ventricular posterior wall (LVPW) thickness (Figure 3E) consistent with ventricular thinning, a typical feature of DCM. Diastolic dysfunction was also consistently present at 56 days ISO + 5-FU when compared to ISO and the CTRL (Figure 3F–H, Table 2). The speckle-tracking based strain analysis showed a persistent global and regional contractility depression at 56 days in ISO + 5-FU mice when compared to the ISO and CTRL animals (Figure 3I–J, Table 2). Finally, a color Doppler analysis of mitral valve demonstrated a persistent ventricular dilation with secondary functional mitral and tricuspid regurgitation in ISO + 5-FU mice at 56 days, which was absent in the ISO and CTRL animals (Figure 3K).

These data conclusively show that ISO + 5-FU regime produces functional cardiac decompensation that models a non-genetic phenotype of DCM.

### 3.4. ISO + 5-FU Administration Causes Cardiomyocyte Remodeling Typical of Dilated Cardiomyopathy

**Figure 4:**
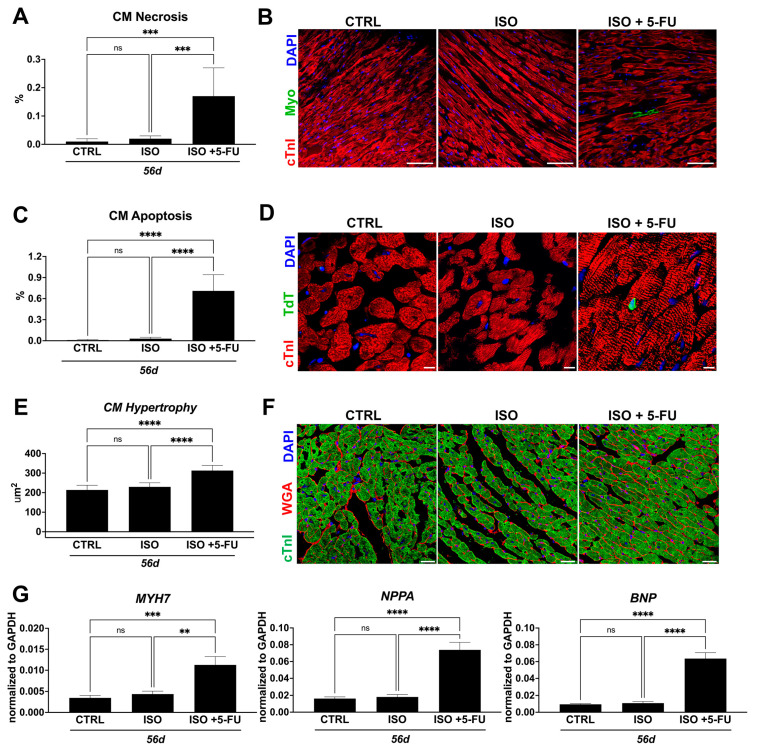
*ISO + 5–FU administration causes cardiomyocyte remodeling typical of dilated cardiomyopathy.* (**A**,**B**) Bar graph with cumulative data (**A**) and relative representative confocal images of cardiac cross-sections (**B**) showing significant cardiomyocyte (CM) necrosis (revealed by myosin-MF20- in vivo antibody labeling) in ISO + 5-FU (n = 3) when compared to CTRL (n = 3) and ISO (n = 3) (MF20, green fluorescence; cTnI, red; DAPI, blue nuclei). Scale bar = 75 µm. *** *p* < 0.001. (**C**,**D**) Bar graph with cumulative data (**C**) and relative representative confocal images of cardiac cross-sections (**D**) showing significant CM apoptosis (assessed by TdT assay) in ISO + 5-FU (n = 5) when compared to CTRL (n = 5) and ISO (n = 5) (TdT, green; cTnI, red; DAPI, blue nuclei). Scale bar = 8 µm. **** *p* < 0.0001. (**E**,**F**) Bar graph with cumulative data (**E**) and relative representative confocal images of cardiac cross-sections (**F**) showing significant CM hypertrophy (assessed by WGA assay) in ISO + 5-FU (n = 5) when compared to CTRL (n = 5) and ISO (n = 5) mice. (WGA, wheat germ agglutinin, red; cTnI, green; DAPI, blue nuclei). Scale bar = 25 µm. **** *p* < 0.0001. (**G**) Bar graphs showing the expressions of stress/hypertrophy genes in cardiomyocytes isolated from ISO + 5-FU (n = 3) when compared to CTRL (n = 3) and ISO (n = 3) mice at 56 days. ** *p* < 0.01; *** *p* < 0.001; **** *p* < 0.0001. ns, no significance.

The ISO high dose administration produces acute cardiomyocyte necrosis and apoptosis [10] that progressively disappear to being indistinguishable from the CTRL at 56 days (Figure 4). On the other hand, cardiomyocyte death (necrosis and apoptosis) is still increased at 56 days in ISO + 5-FU mice (Figure 4). Indeed at 56 days, myosin-labelled necrotic CMs (measured by in vivo myosin antibody labelling) were 0.17 ± 0.1%, respectively, when compared to 0.02 ± 0.01% in ISO and 0.02 ± 0.01% in the CTRL mice (Figure 4A,B). The number of apoptotic cardiomyocytes (identified by TdT labelling) in the CTRL and ISO animals was minimal at 56 days (0.01 ± 0.01% and 0.03 ± 0.02%, respectively) (Figure 4C,D). On the contrary, TdT positive apoptotic cardiomyocytes in the LV of ISO + 5-FU-treated animals were significantly increased at 56 days (0.71 ± 0.23%) (Figure 4C,D). Cardiomyocyte drop out in ISO + 5-FU mice was associated with evident hypertrophy (cardiomyocyte size = 313 ± 26 μm^2^) in ISO + 5-FU mice when compared to ISO (cardiomyocyte size = 230 ± 21 μm^2^) and the CTRL (cardiomyocyte size = 214 ± 24 μm^2^) (Figure 4E,F).

A CM hypertrophic response in ISO + 5-FU mice was confirmed by a ~3-, 5-, and -7-fold increase in ANF, BNP, and MYH7 gene expression, respectively, in isolated cardiomyocytes from an additional 3 mice at 56 days when compared to the CTRL and ISO (Figure 4G)

These data show that the ISO + 5-FU regime produces chronic cardiomyocyte death ensuing in a stress hypertrophic cardiomyocyte response typical of dilated cardiomyopathy.

### 3.5. Acute Isoproterenol Exposure Followed by 5-Fluorouracil Administration Causes Myocardial Tissue Alterations Typical of Dilated Cardiomyopathy

**Figure 5:**
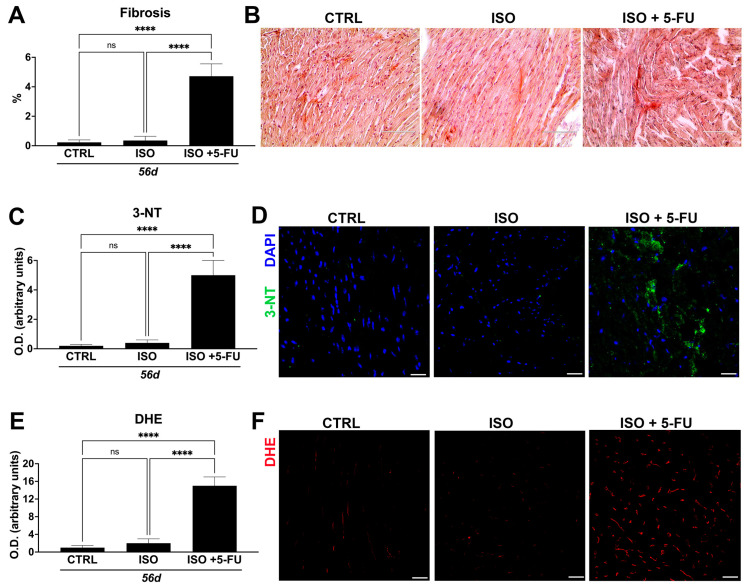
*Acute isoproterenol exposure followed by 5–FU administration causes myocardial tissue alterations typical of DCM.* (**A**,**B**) Bar graph with cumulative data (**A**) and relative representative light microscopy images of cardiac cross-sections (**B**) showing significant myocardial fibrosis (revealed by Picrosirius Red staining) and cardiomyocyte disarray in ISO + 5-FU (n = 5) when compared to CTRL (n = 5) and ISO (n = 5) (cTnI, red; DAPI, blue nuclei). **** *p* < 0.0001. (**C**,**D**) Bar graph with cumulative data (**C**) and relative representative confocal images of cardiac cross-sections (**D**) showing exaggerated myocardial tissue ROS production (assessed by Nitrotyrosine, 3-NT) in ISO + 5-FU (n = 5) when compared to CTRL (n = 5) and ISO (n = 5) (3-NT, green; DAPI, blue nuclei). Scale bar = 25 µm. **** *p* < 0.0001. (**E**,**F**) Bar graph with cumulative data (**E**) and relative representative confocal images of cardiac cross-sections (**F**) showing exaggerated myocardial tissue ROS production (assessed by Dihydroethidium, DHE) in ISO + 5-FU (n = 5) when compared to CTRL (n = 5) and ISO (n = 5) mice. (DHE, red). Scale bar = 25 µm. **** *p* < 0.0001. ns, no significance.

Cardiomyocyte chronic damage as produced by ISO + 5-FU regime postulated the presence of reactive fibrosis as another element typical of cardiac remodeling of dilated cardiomyopathy. Accordingly, the level of fibrosis captured and quantified by picrosirius red staining of myocardial sections from ISO + 5-FU, ISO and CTRL hearts revealed significantly elevated fibrosis with cardiomyocyte disarray at 56 days in hearts from ISO + 5-FU animals (5 ± 1%) compared to ISO (0.4 ± 0.2) and CTRL (0.2 ± 0.1) hearts (Figure 5A,B).

Over time, progressive deterioration of contractile function perpetuates myocyte damage, which may stimulate oxidative stress that aggravates the progression of cardiomyopathies [16,17]. As we observed severe cardiac dysfunction, myocyte damage, and fibrosis, we next determined whether oxidative stress was elevated in the hearts of ISO + 5-FU mice. Cardiac samples were thus analyzed for the presence of nitrotyrosine (3-NT), and dihydroethidium (DHE). ISO + 5-FU resulted in an exacerbated increase (of ~5- and 10-fold, respectively) of myocardial cells positive for these markers of oxidative stress when compared to the CTRL and ISO (Figure 5C–F).

Overall, these data indicate a scar tissue development and loss of structural integrity accompanied by increased ROS in ISO + 5-FU hearts, histopathological features typical of dilated cardiomyopathy.

### 3.6. ISO + 5-FU Administration Produces Myocardial Tissue Inflammation Associated with Senescence Accumulation

**Figure 6:**
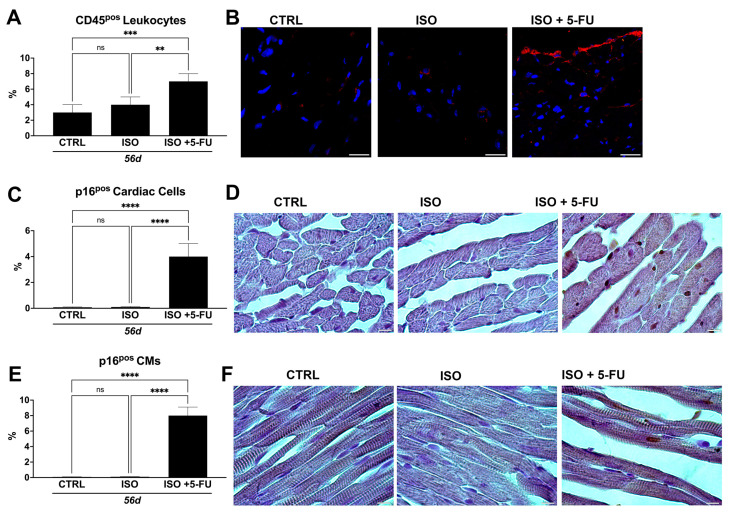
*ISO + 5–FU administration produces myocardial tissue inflammation associated with senescence accumulation.* (**A**,**B**) Bar graph with cumulative data (**A**) and relative representative confocal microscopy images of cardiac cross-sections (**B**) showing increased inflammatory cells (revealed by leukocyte marker, CD45, staining) in ISO + 5-FU (n = 5) when compared to CTRL (n = 5) and ISO (n = 5) (CD45, red; DAPI, blue nuclei). ** *p* < 0.001; *** *p* < 0.0001. Scale bar = 25 µm. (**C**,**D**) Bar graph with cumulative data (**C**) and relative representative light microscopy images of cardiac cross-sections (**D**) showing accumulation of senescent cardiac cells (assessed by p16 antibody staining) in ISO + 5-FU (n = 5) when compared to CTRL (n = 5) and ISO (n = 5) (p16, brown DAB staining). **** *p* < 0.0001. Scale bar = 10 µm. (**E**,**F**) Bar graph with cumulative data (**E**) and relative representative light microscopy images of cardiac cross-sections (**F**) showing accumulation of senescent CMs (assessed by p16 antibody staining)) in ISO + 5-FU (n = 5) when compared to CTRL (n = 5) and ISO (n = 5) (p16, brown DAB staining). **** *p* < 0.0001. Scale bar = 10 µm. ns, no significance.

CD45^+^ leukocytes increased significantly on days 56 in ISO + 5-FU mice (7 ± 1%) when compared to hearts from the CTRL (3 ± 1%) and ISO (4 ± 1%) mice (Figure 6A,B). Inflammation was dispersed throughout the myocardium.

As an inflammatory response is one of the hallmarks of cellular senescence [18,19], we evaluated senescence in the ISO + 5-FU model by assessing p16 expression, a typical marker of cell senescence [20]. Intriguingly, at 56 days from the primary insult, the hearts from the ISO + 5-FU group showed a significant increase in p16 positive (pos) cardiac cells (4 ± 1%) when compared to the CTRL (0.08 ± 0.03%) and ISO (0.1 ± 0.04%) groups (Figure 6C,D). Additionally, despite the fact that cardiomyocytes are terminally differentiated cells, they also can acquire a senescent-like phenotype in cardiac disease [11,21]. Accordingly, the ISO + 5-FU regime significantly increased p16^pos^ cardiomyocytes (8 ± 2%) when compared to the CTRL (0.05 ± 0.03%) and ISO (0.09 ± 0.02%) groups (Figure 6E,F).

Overall, these data show that ISO + 5-FU produces an inflammatory cardiac milieu that reproduces the pathophysiological process of DCM.

## 4. Discussion

The main findings emanating from this study are: (i) ISO + 5-FU in mice induces progressive ventricular dilation and reduced systolic function, along with diastolic dysfunction; (ii) a 5-FU administration upon acute damage by ISO transformed a transient and mostly segmental cardiac decompensation by ISO alone (a Takotsubo-like cardiomyopathy) into a global and diffuse cardiac contractility depression; (iii) the ISO + 5-FU regime produces persistent functional cardiac decompensation; (iv) the ISO + 5-FU regime produces chronic cardiomyocyte death ensuing in a stress hypertrophic cardiomyocyte response typical of dilated cardiomyopathy; (v) scar tissue development and a loss of structural integrity are accompanied by increased ROS in ISO + 5-FU hearts; (vi) ISO + 5-FU produces a chronic myocardial inflammatory reaction with premature cell senescence.

DCM is a condition in which the heart muscle becomes weakened and enlarged owing to heart failure, and it is the most common form of the several existing cardiomyopathy types with a poor prognosis [5,6,22]. To the aim of identifying new therapeutic strategies for improved outcomes, animal models that reproduce the main features of DCM are warranted, and organoids are going to be fundamental as pre-clinical tests on human cells/tissues [23].

Both acute and chronic models of ISO have been used to study cardiomyopathies [24,25]. Acute exposure to a single overdose of ISO mimics stress-induced cardiomyopathy [19,26,27]. In contrast, the chronic model uses an implanted minipump to release ISO continuously. This model mimics advanced heart failure where there is chronic adrenergic stimulation [28]. In particular, chronic exposure to Isoproterenol is a pharmacological and pathological stimuli in vivo to produce a model of hypertrophic cardiomyopathy [29]. Cardiotoxicity from chemotherapy, like anthracyclines, has been used to study chronic cardiomyopathy [30,31]. 5-FU is the second most common chemotherapeutic drug associated with cardiotoxicity after anthracyclines, which can manifest as chest pain, acute coronary syndrome/myocardial infarction, or death [32]. We and others have previously and reproducibly shown that an acute exposure to a single high dose of Isoproterenol (ISO) produces reversible myocardial injury with cardiomyocyte dropout and transient cardiac dysfunction in mice [8,9,10,15]. Indeed, a subcutaneous injection of 200 mg/kg of ISO in male C57BL/6J mice produces diffuse cardiomyocyte death at one day followed by an LV ejection fraction (EF) and fractional shortening (FS) depression accompanied by diastolic dysfunction [10]. ISO-dependent damage activates the resident cardiac stem/progenitor cells (CSCs) generating a burst of new cardiomyocyte formation that replaces those lost to ISO, restoring normal cardiac contractility [9,10,15,33]. Importantly, all the morphological and functional parameters return to baseline at 28 days after ISO [10]. We have also shown that when ISO administration is followed by the administration of low doses of the anti-mitotic agent 5-FU, there is no recovery from acute histological and functional damage, resulting in a severe cardiomyopathy over long term [10,15]. Indeed, when ISO is followed by chronic exposure to low doses of 5-FU, the endogenous reparative response is abolished with no functional recovery [10,15]. The regenerative process is completely restored by replacing the ablated CSCs with the progeny of one CSC [10,15]. We have already shown that these effects are not due to 5-FU cardiotoxicity because the same 5-FU regime administered to control animals (not treated with ISO) does not cause any functional or histological cardiac toxic effects [15]. We therefore evaluated the ISO + 5-FU regime as a model of DCM.

Remarkably, ISO + 5-FU causes persistent cardiomyocyte damage and cardiomyocyte death by necrosis and apoptosis in a myocardium characterized by inflammation, ROS increase and fibrosis. Interestingly, cardiomyocyte necrosis and apoptosis were diffusely distributed from epi to endocardial layers and from base to apex in ISO + 5-FU mice when compared to the mostly subendocardial apex damage after acute ISO [10]. These cellular alterations underlie anatomical and functional cardiac changes with progressive LV dilation and severe cardiac decompensation. Cardiac dilation is accompanied by both mitral and tricuspid regurgitation, indicative of biventricular dysfunction. In addition, a strain analysis revealed severe myocardial contractility depressions, and tissue doppler showed persistent diastolic disfunction. These results demonstrate that 5-FU administration upon acute damage by ISO transformed a transient and mostly segmental cardiac decompensation by ISO alone (a Takotsubo-like cardiomyopathy) into a persistent global and diffuse cardiac contractility depression typical of dilated cardiomyopathy in mice.

An ISO-induced myocardial injury acutely activates inflammation and cardiac resident innate immune cells, which return to baseline overtime [34,35]. The administration of 5-FU following ISO overdose abolishes the inflammatory response that fails to raise acutely after ISO damage [15]. However, an inflammatory response sets up over long term, establishing low grade inflammation in the DCM induced by ISO + 5-FU. Oxidative stress has been demonstrated to be elevated in human hypertrophic CM (HCM), as well as DCM caused by viral infection, inflammation, toxic substances, and mutation of cTnT [16,17]. Of interest, cardiac damage, oxidative stress, and an inflammatory response by ISO + 5-FU ensue in exacerbated cardiac cell senescence of both non-myocyte and myocyte cells, a trait attributable to the so-called pattern of ‘inflammaging’ [14,20,21]. A senescence–inflammatory response is indeed key in cardiac and aging-cardiac related diseases [20,21]. When considering that DCM accumulates inflammation with age and that DCM has a progressively worst prognosis with age, it is tempting to speculate that DCM is the product of a tissue microenvironment of premature aging, similar to diabetic cardiomyopathy [14,36,37], secondary to ongoing cell death, inflammation, and cell senescence, which decrease/abolish the cardiac regenerative/reparative capacity with progressive cardiac dysfunction with age. Of course, the latter requires further study to be experimentally demonstrated.

Finally, it should be noted that, here, 5-FU is used to abrogate the intrinsic endogenous reparative/regenerative capacity of the adult heart, which restores anatomy and function upon an ISO-induced injury [10,15]. Therefore, 5-FU is not intended to create direct myocardial injury, but to prevent the cellular mechanisms mediating recovery from ISO-dependent myocardial injury. 5-FU specifically ablates the activation of endogenous cardiac progenitors and removes the inflammatory response acutely after ISO administration. 5-FU is administered through osmotic pumps to have a systemic constant release of low doses of the drug that can target only highly proliferative cells. The latter explains why in intact animals (not injured by ISO) these low doses do not injury the myocardium, which is a tissue with a very low proliferative activity in physiological states, as indeed cardiomyocytes are terminally differentiated cells and non-myocyte cells are mostly kept in a quiescent state in physiological conditions. Overall, the injury is created by Isoproterenol, and then, 5-FU blunts the reparative and regenerative response ensuing in a chronic cardiomyopathy with ongoing cell death, chronic inflammation, and a lack of regenerative capacity with anatomical and functional resemblance of DCM.

## 5. Conclusions

The present findings show that a combination of Isoproterenol plus 5-FU produced anatomical, histological, and functional cardiac alterations typical of DCM, representing a widely available, affordable, and reproducible mouse model of this cardiomyopathy.

## Figures and Tables

**Table 1:** Echocardiographic data from baseline to 28 days *.

	Baseline (n = 26)	Ctrl 2d (n = 8)	ISO 2d (n = 18)	Ctrl 14d (n = 8)	ISO 14d (n = 8)	ISO + 5-FU 14d (n = 8)	Ctrl 28d (n = 8)	ISO 28d (n = 8)	ISO + 5-FU 28d (n = 8)
**LVEDD (mm)**	3.82 ± 0.24	3.9 ± 0.33	4.22 ± 0.41	3.91 ± 0.16	3.95 ± 0.14	4.3 ± 0.14	3.9 ± 0.1	3.92 ± 0.21	4.35 ± 0.2
**LVESD (mm)**	2.63 ± 0.25	2.62 ± 0.4	3.23 ± 0.38	2.64 ± 0.14	2.69 ± 0.16	3.23 ± 0.19	2.62 ± 0.13	2.7 ± 0.35	3.48 ± 0.12
**LVPWd (mm)**	0.75 ± 0.07	0.76 ± 0.05	0.8 ± 0.1	0.78 ± 0.04	0.78 ± 0.09	0.71 ± 0.04	0.79 ± 0.07	0.79 ± 0.04	0.68 ± 0.03
**EF (%)**	60.7 ± 3.92	62.1 ± 6.5	48.05 ± 2.92	59.62 ± 2.99	58.51 ± 3.28	48.47 ± 3.73	61.9 ± 5.35	59.5 ± 2.75	39.9 ± 4.53
**FS (%)**	31.15 ± 2.63	32.82 ± 6.4	23.46 ± 1.5	32.48 ± 2.04	31.9 ± 2.26	24.59 ± 2.49	32.82 ± 3.87	31.12 ± 1.8	20 ± 4
**MV E (mm/s)**	705.57 ± 103.14	691.6 ± 74.44	718.62 ± 97.67	701.65 ± 111.74	734.07 ± 92.78	803.01 ± 66.79	700.18 ± 72.42	750.07 ± 40.01	756.11 ± 99.85
**E′ (mm/s)**	26.79 ± 4.23	26.51 ± 3.37	20.66 ± 4.16	27.38 ± 1.86	25.99 ± 3.14	22.6 ± 4.01	26.97 ± 3.65	27.55 ± 4.17	20.18 ± 1.55
**E/E′**	26.72 ± 4.33	26.4 ± 3.96	35.32 ± 3.8	25.6 ± 25.6	28.45 ± 4.02	36.19 ± 5.2	26.25 ± 3.41	27.73 ± 3.23	37.58 ± 4.98
**GLS (%)**	−20.01 ± 2.83	−19.64 ± 2.66	−13.25 ± 1.9	−20.33 ± 3.32	−19.31 ± 2.42	−15.12 ± 3.21	−19.74 ± 2.49	−19.06 ± 2.62	−12.7 ± 2.58
**GCS (%)**	−22.83 ± 2.06	−21.49 ± 4.35	−20.15 ± 3.8	−22.53 ± 4.38	−21.92 ± 3.42	−19.36 ± 5.04	−21.82 ± 4.13	−20.73 ± 3.44	−17.72 ± 6.67

* Statistical significance is reported in Figure 1 and Figure 2.

**Table 2:** Echocardiographic data at 56 days *.

	Ctrl 56d (n = 8)	ISO 56d (n = 8)	ISO + 5-FU 56d (n = 8)
**LVEDD (mm)**	3.93 ± 0.11	3.92 ± 0.15	4.38 ± 0.21
**LVESD (mm)**	2.59 ± 0.08	2.72 ± 0.18	3.51 ± 0.27
**LVPWd (mm)**	0.79 ± 0.085	0.8 ± 0.09	0.65 ± 0.04
**EF (%)**	63.61 ± 4.55	60.25 ± 5.95	40.61 ± 3.39
**FS (%)**	33.98 ± 3.48	30.57 ± 4.45	18.8 ± 3.35
**MV E (mm/s)**	672.8 ± 74	714.68 ± 77.18	725.26 ± 77.5
**E′ (mm/s)**	25.72 ± 4.59	26.25 ± 3.6	19.1 ± 1.92
**E/E′**	26.39 ± 4.92	27.65 ± 3.14	38.26 ± 5.08
**GLS (%)**	−19.62 ± 2.56	−19.48 ± 3.17	−12.44 ± 3.55
**GCS (%)**	−23.79 ± 3.38	−23.25 ± 3.19	−17.56 ± 2.15

* Note: Statistical significance is reported in Figure 3.

## Data Availability

All data are available within this article.
